# Synthesizing Context-free Grammars from Recurrent Neural Networks

**DOI:** 10.1007/978-3-030-72016-2_19

**Published:** 2021-03-01

**Authors:** Daniel M. Yellin, Gail Weiss

**Affiliations:** 8 Eindhoven University of Technology, Eindhoven, The Netherlands; 9 Aalborg University, Aalborg East, Denmark; 10 IBM, Givatayim, Israel; 11 Technion, Haifa, Israel

**Keywords:** Model Extraction, Learning Context Free Grammars, Finite State Machines, Recurrent Neural Networks

## Abstract

We present an algorithm for extracting a subclass of the context free grammars (CFGs) from a trained recurrent neural network (RNN). We develop a new framework, *pattern rule sets* (PRSs), which describe sequences of deterministic finite automata (DFAs) that approximate a non-regular language. We present an algorithm for recovering the PRS behind a sequence of such automata, and apply it to the sequences of automata extracted from trained RNNs using the $L^{*}$ algorithm. We then show how the PRS may converted into a CFG, enabling a familiar and useful presentation of the learned language.

Extracting the learned language of an RNN is important to facilitate understanding of the RNN and to verify its correctness. Furthermore, the extracted CFG can augment the RNN in classifying correct sentences, as the RNN’s predictive accuracy decreases when the recursion depth and distance between matching delimiters of its input sequences increases.

## 1 Introduction

Recurrent Neural Networks (RNNs) are a class of neural networks adapted to sequential input, enjoying wide use in a variety of sequence processing tasks. Their internal process is opaque, prompting several works into extracting interpretable rules from them. Existing works focus on the extraction of deterministic or weighted finite automata (DFAs and WFAs) from trained RNNs [18,6,26,3].

However, DFAs are insufficient to fully capture the behavior of RNNs, which are known to be theoretically Turing-complete [20], and for which there exist architecture variants such as LSTMs [14] and features such as stacks [9,23] or attention [4] increasing their practical power. Several recent investigations explore the ability of different RNN architectures to learn Dyck, counter, and other non-regular languages [19,5,28,21], with mixed results.

While the data indicates that RNNs can generalize and achieve high accuracy, they do not learn hierarchical rules, and generalization deteriorates as the length and 'depth' of the input grows [19,5,28]. Sennhauser and Berwick conjecture that

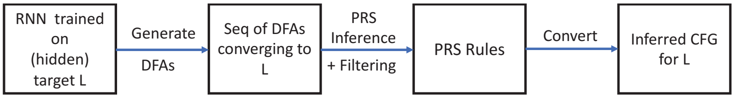

**Fig. 1.** Overview of steps in algorithm to synthesize the hidden language $L$

"what the LSTM has in fact acquired is sequential statistical approximation to this solution" instead of "the 'perfect' rule-based solution" [19]. Similarly, Yu et. al. conclude that "the RNNs can not truly model CFGs, even when powered by the attention mechanism" [28]. This is line with Hewitt et. al., who note that a fixed precision RNN can only learn a language of fixed depth strings [13].

*Goal of this paper* We wish to extract a CFG from a trained RNN. In particular, we wish to find the CFG that not only explains the finite language learnt by the RNN, but generalizes it to strings of unbounded depth and distance.

*Our approach* Our method builds on the DFA extraction work of Weiss et al. [26], which uses the $L^*$ algorithm [2] to learn the DFA of a given RNN. As part of the learning process, $L^*$ creates a sequence of *hypothesis DFAs* approximating the target language. Our main insight is in treating these hypothesis DFAs as coming from a set of underlying rules, that recursively improve each DFA's approximation of the target CFG by increasing the distance and embedded depth of the sequences it can recognize. In this light, synthesizing the target CFG becomes the problem of recovering these rules.

We propose the framework of *pattern rule sets* (PRSs) for describing such rule applications, and present an algorithm for recovering a PRS from a sequence of DFAs. We also provide a method for converting a PRS to a CFG, and test our method on RNNs trained on several PRS languages. Pattern rule sets are expressive enough to cover several variants of the Dyck languages, which are prototypical context-free languages (CFLs): the Chomsky–Schützenberger representation theorem shows that any CFL can be expressed as a homomorphic image of a Dyck language intersected with a regular language[16].

A significant issue we address is that the extracted DFAs are often inexact, either through inaccuracies in the RNN, or as an artifact of the $L^*$ algorithm.

To the best of our knowledge, this is the first work on synthesizing a CFG from a general RNN (though some works extract push-down automata [23,9] from RNNs with an external stack, they do not apply to plain RNNs). The overall steps in our technique are given in Figure 1.

*Contributions* The main contributions of this paper are:

– *Pattern Rule Sets* (PRSs), a framework for describing a sequence of DFAs approximating a CFL.
– An algorithm for recovering the PRS generating a sequence of DFAs, that may also be applied to noisy DFAs elicited from an RNN using $L^*$ .
– An algorithm converting a PRS to a CFG.

– An implementation of our technique[1], and an evaluation of its success on recovering various CFLs from trained RNNs.

## 2 Definitions and Notations

### 2.1 Deterministic Finite Automata

**Definition 1 (Deterministic Finite Automata).** *A deterministic finite automaton (DFA) over an alphabet* $\Sigma$ *is a 5-tuple* $\langle \Sigma, q_0, Q, F, \delta \rangle$ *such that* $Q$ *is a finite set of states,* $q_0 \in Q$ *is the initial state,* $F \subseteq Q$ *is a set of final (accepting) states and* $\delta : Q \times \Sigma \to Q$ *is a (possibly partial) transition function.*

Unless stated otherwise, we assume each DFA's states are unique to itself, i.e., for any two DFAs $A, B$ – including two instances of the same DFA – $Q_A \cap Q_B = \emptyset$. A DFA $A$ is said to be *complete* if $\delta$ is complete, i.e., the value $\delta(q, \sigma)$ is defined for every $q, \sigma \in Q \times \Sigma$. Otherwise, it is *incomplete*.

We define the extended transition function $\hat{\delta} : Q \times \Sigma^* \to Q$ and the language $L(A)$ accepted by $A$ in the typical fashion. We also associate a language with intermediate states of $A$: $L(A, q_1, q_2) \triangleq \{w \in \Sigma^* \mid \hat{\delta}(q_1, w) = q_2\}$. The states from which no sequence $w \in \Sigma^*$ is accepted are known as the *sink reject states*.

**Definition 2.** *The* sink reject states *of a DFA* $A = \langle \Sigma, q_0, Q, F, \delta \rangle$ *are the maximal set* $Q_R \subseteq Q$ *satisfying:* $Q_R \cap F = \emptyset$*, and for every* $q \in Q_R$ *and* $\sigma \in \Sigma$*, either* $\delta(q, \sigma) \in Q_R$ *or* $\delta(q, \sigma)$ *is not defined.*

**Definition 3 (Defined Tokens).** *Let* $A = \langle \Sigma, q_0, Q, F, \delta \rangle$ *be a complete DFA with sink reject states* $Q_R$*. For every* $q \in Q$*, its* defined tokens *are* $\mathrm{def}(A, q) \triangleq \{\sigma \in \Sigma \mid \delta(q, \sigma) \notin Q_R\}$*. When the DFA* $A$ *is clear from context, we write* $\mathrm{def}(q)$*.*

All definitions for complete DFAs are extended to incomplete DFAs $A$ by considering their *completion* - an extension of $A$ in which all missing transitions are connected to a (possibly new) sink reject state.

**Definition 4 (Set Representation of $\delta$).** *A (possibly partial) transition function* $\delta : Q \times \Sigma \to Q$ *may be equivalently defined as the set* $S_\delta = \{(q, \sigma, q') \mid \delta(q, \sigma) = q'\}$*. We use* $\delta$ *and* $S_\delta$ *interchangeably.*

**Definition 5 (Replacing a State).** *For a transition function* $\delta : Q \times \Sigma \to Q$*, state* $q \in Q$*, and new state* $q_n \notin Q$*, we denote by* $\delta_{[q \leftarrow q_n]} : Q' \times \Sigma \to Q'$ *the transition function over* $Q' = (Q \setminus \{q\}) \cup \{q_n\}$ *and* $\Sigma$ *that is identical to* $\delta$ *except that it redirects all transitions into or out of* $q$ *to be into or out of* $q_n$*.*

[1] The implementation for this paper, and a link to all trained RNNs, is available at `https://github.com/tech-srl/RNN_to_PRS_CFG`.

### 2.2 Dyck Languages

A Dyck language *of order N* is expressed by the grammar `D ::=` $\varepsilon$ `|` $\mathtt{L}_1$ `D` $\mathtt{R}_1$ `| ... |` $\mathtt{L}_N$ `D` $\mathtt{R}_N$ `| D D` with unique symbols $\mathtt{L}_1,\ldots,\mathtt{L}_N,\mathtt{D}_1,\ldots,\mathtt{D}_N$. A common measure of complexity for a Dyck word is its maximum *distance* (number of characters) between matching delimiters and *embedded depth* (number of unclosed delimiters) [19]. We generalize and refer to *Regular Expression Dyck (RE-Dyck)* languages as languages expressed by the same CFG, except that each $\mathtt{L}_i$ and each $\mathtt{R}_i$ derive some regular expression.

We present regular expressions as is standard, for example: $L(\{\mathtt{a|b}\}\cdot\mathtt{c}) \triangleq \{\mathtt{ac,bc}\}$.

## 3 Patterns

Patterns are DFAs with a single *exit* state $q_X$ in place of a set of final states, and with no cycles on their initial or exit states unless $q_0 = q_X$.

**Definition 6 (Patterns).** *A* pattern $p = \langle \Sigma, q_0, Q, q_X, \delta\rangle$ *is a DFA* $A^p = \langle \Sigma, q_0, Q, \{q_X\}, \delta\rangle$*, satisfying: 1.* $L(A^p) \neq \emptyset$*, and 2. either* $q_0 = q_X$*, or* $\mathrm{def}(q_X) = \emptyset$ *and* $L(A, q_0, q_0) = \{\varepsilon\}$*. If* $q_0 = q_X$ *then p is called* circular*, otherwise, it is* non-circular*. Patterns are always given in minimal incomplete presentation.*

We refer to a pattern's initial and exit states as its *edge states*. All the definitions for DFAs apply to patterns through $A^p$. We denote each pattern $p$'s language $L_p \triangleq L(p)$, and if it is marked by some superscript $i$, we refer to all of its components with superscript $i$: $p^i = \langle \Sigma, q_0^i, Q^i, q_X^i, \delta^i\rangle$.

### 3.1 Pattern Composition

We can compose two non-circular patterns $p^1, p^2$ by merging the exit state of $p^1$ with the initial state of $p^2$, creating a new pattern $p^3$ satisfying $L_{p^3} = L_{p^1}\cdot L_{p^2}$.

**Definition 7 (Serial Composition).** *Let* $p^1, p^2$ *be two non-circular patterns. Their* serial composite *is the pattern* $p^1 \circ p^2 = \langle \Sigma, q_0^1, Q, q_X^2, \delta\rangle$ *in which* $Q = Q^1 \cup Q^2 \setminus \{q_X^1\}$ *and* $\delta = \delta^1_{[q_X^1 \leftarrow q_0^2]} \cup \delta^2$*. We call* $q_0^2$ *the* join state *of this operation.*

If we additionally merge the exit state of $p_2$ with the initial state of $p_1$, we obtain a circular pattern $p$ which we call the *circular composition* of $p_1$ and $p_2$. This composition satisfies $L_p = \{L_{p_1}\cdot L_{p_2}\}^*$.

**Definition 8 (Circular Composition).** *Let* $p^1, p^2$ *be two non-circular patterns. Their* circular composite *is the circular pattern* $p_1 \circ_c p_2 = \langle \Sigma, q_0^1, Q, q_0^1, \delta\rangle$ *in which* $Q = Q^1 \cup Q^2 \setminus \{q_X^1, q_X^2\}$ *and* $\delta = \delta^1_{[q_X^1 \leftarrow q_0^2]} \cup \delta^2_{[q_X^2 \leftarrow q_0^1]}$*. We call* $q_0^2$ *the* join state *of this operation.*

Figure 2 shows 3 examples of serial and circular compositions of patterns.

Patterns do not carry information about whether or not they have been composed from other patterns. We maintain such information using *pattern pairs*.

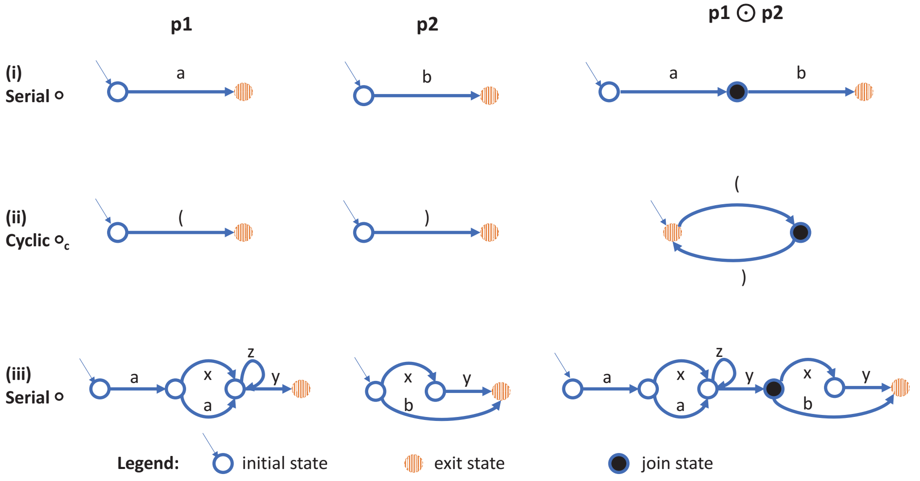

**Fig. 2.** Examples of the composition operator

**Definition 9 (Pattern Pair).** *A* pattern pair *is a pair* $\langle P, P_c \rangle$ *of pattern sets, such that* $P_c \subset P$ *and for every* $p \in P_c$ *there exists exactly one pair* $p_1, p_2 \in P$ *satisfying* $p = p_1 \odot p_2$ *for some* $\odot \in \{\circ, \circ_c\}$*. We refer to the patterns* $p \in P_c$ *as the* composite patterns *of* $\langle P, P_c \rangle$*, and to the rest as its* base patterns*.*

We will often discuss patterns that have been composed into larger DFAs.

**Definition 10 (Pattern Instances).** *Let* $A = \langle \Sigma, q_0^A, Q^A, F, \delta^A \rangle$ *be a DFA,* $p = \langle \Sigma, q_0, Q, q_X, \delta \rangle$ *be a pattern, and* $\hat{p} = \langle \Sigma, q_0', Q', q_X', \delta' \rangle$ *be a pattern 'inside'* $A$*, i.e.,* $Q' \subseteq Q^A$ *and* $\delta' \subseteq \delta^A$*. We say that* $\hat{p}$ *is an* instance of $p$ in $A$ *if* $\hat{p}$ *is isomorphic to* $p$*.*

A pattern instance in a DFA $A$ is uniquely determined by its structure and initial state: $(p, q)$. If $p$ is a composite pattern with respect to some pattern pair $\langle P, P_c \rangle$, the join state of its composition within $A$ is also uniquely defined.

**Definition 11.** *For every pattern pair* $\langle P, P_c \rangle$*, for each composite pattern* $p \in P_c$*, DFA* $A$*, and initial state* $q$ *of an instance* $\hat{p}$ *of* $p$ *in* $A$*,* $\text{join}(p, q, A)$ *returns the join state of* $\hat{p}$ *with respect to its composition in* $\langle P, P_c \rangle$*.*

## 4 Pattern Rule Sets

For any infinite sequence $S = A_1, A_2, ...$ of DFAs satisfying $L(A_i) \subset L(A_{i+1})$, for all $i$, we define the language of $S$ as the union of the languages of all these DFAs: $L(S) = \cup_i L(A_i)$. Such sequences may be used to express CFLs.

In this work we take a finite sequence $A_1, A_2, ..., A_n$ of DFAs, and assume it is a (possibly noisy) finite prefix of an infinite sequence of approximations for a language, as above. We attempt to reconstruct the language by guessing how the sequence may continue. To allow such generalization, we must make assumptions about how the sequence is generated. For this we introduce *pattern rule sets*.

Pattern rule sets (PRSs) create sequences of DFAs with a single accepting state. Each PRS is built around a pattern pair $\langle P, P_c \rangle$, and each rule application connects a new pattern instance to the current DFA $A_i$, at the join state of a composite-pattern inserted into $A_i$ at some earlier point. To define where a pattern can be connected to $A_i$, we introduce an *enabled instance* set $\mathcal{I}$.

**Definition 12.** *An* enabled DFA *over a pattern pair* $\langle P, P_c \rangle$ *is a tuple* $\langle A, \mathcal{I} \rangle$ *such that* $A = \langle \Sigma, q_0, Q, F, \delta \rangle$ *is a DFA and* $\mathcal{I} \subseteq P_c \times Q$ *marks* enabled instances *of composite patterns in* $A$.

Intuitively, for every enabled DFA $\langle A, \mathcal{I} \rangle$ and $(p, q) \in \mathcal{I}$, we know: (i) there is an instance of pattern $p$ in $A$ starting at state $q$, and (ii) this instance is *enabled*; i.e., we may connect new pattern instances to its join state $\text{join}(p, q, A)$.

**Definition 13.** *A PRS* $\mathbf{P}$ *is a tuple* $\langle \Sigma, P, P_c, R \rangle$ *where* $\langle P, P_c \rangle$ *is a pattern pair over the alphabet* $\Sigma$ *and* $R$ *is a set of* rules. *Each rule has one of the following forms, for some* $p, p^1, p^2, p^3, p^I \in P$, *with* $p^1$ *and* $p^2$ *non-circular:*

(1) $\bot \twoheadrightarrow p^I$
(2) $p \twoheadrightarrow_c (p^1 \odot p^2) \eqcirc p^3$, *where* $p = p^1 \odot p^2$ *for* $\odot \in \{\circ, \circ_c\}$, *and* $p^3$ *is circular*
(3) $p \twoheadrightarrow_s (p^1 \circ p^2) \eqcirc p^3$, *where* $p = p^1 \circ p^2$ *and* $p^3$ *is non-circular*

A PRS derives sequences of enabled DFAs as follows: first, a rule of type (1) creates $\langle A_1, \mathcal{I}_1 \rangle$ according to $p^I$. Then, for every $\langle A_i, \mathcal{I}_i \rangle$, each rule may connect a new pattern instance to $A_i$, specifically at a state determined by $\mathcal{I}_i$.

**Definition 14 (Initial Composition).** $\mathcal{D}_1 = \langle A_1, \mathcal{I}_1 \rangle$ *is generated from a rule* $\bot \twoheadrightarrow p^I$ *as follows:* $A_1 = A^{p^I}$, *and* $\mathcal{I}_i = \{(p^I, q_0^I)\}$ *if* $p^I \in P_c$ *and otherwise* $\mathcal{I}_1 = \emptyset$.

Let $\mathcal{D}_i = \langle A_i, \mathcal{I}_i \rangle$ be the enabled DFAat step $i$ and denote $A_i = \langle \Sigma, q_0, Q, F, \delta \rangle$. Note that for $A_1$, $|F| = 1$, and for all $A_{i+1}$, $F$ is unchanged (by future definitions).

Rules of type (1) extend $A_i$ by grafting a circular pattern to $q_0$, and then enabling that pattern if it is composite.

**Definition 15 (Rules of type (1)).** *A rule* $\bot \twoheadrightarrow p^I$ *with circular* $p^I$ *may extend* $\langle A_i, \mathcal{I}_i \rangle$ *at the initial state* $q_0$ *of* $A_i$ *iff* $\text{def}(q_0) \cap \text{def}(q_0^I) = \emptyset$. *This creates the DFA* $A_{i+1} = \langle \Sigma, q_0, Q \cup Q^I \setminus \{q_0^I\}, F, \delta \cup \delta^I_{[q_0^I \leftarrow q_0]} \rangle$. *If* $p^I \in P_c$ *then* $\mathcal{I}_{i+1} = \mathcal{I}_i \cup \{(p^I, q_0)\}$, *else* $\mathcal{I}_{i+1} = \mathcal{I}_i$.

Rules of type (2) graft a circular pattern $p^3 = \langle \Sigma, q_0^3, q_x^3, F, \delta^3 \rangle$ onto the join state $q_j$ of an enabled pattern instance $\hat{p}$ in $A_i$, by merging $q_0^3$ with $q_j$. In doing so, they also enable the patterns composing $\hat{p}$, if they are composite.

**Definition 16 (Rules of type (2)).** *A rule* $p \twoheadrightarrow_c (p^1 \odot p^2) \eqcirc p^3$ *may extend* $\langle A_i, \mathcal{I}_i \rangle$ *at the join state* $q_j = \text{join}(p, q, A_i)$ *of any instance* $(p, q) \in \mathcal{I}_i$, *provided* $\text{def}(q_j) \cap \text{def}(q_0^3) = \emptyset$. *This creates* $\langle A_{i+1}, \mathcal{I}_{i+1} \rangle$ *as follows:* $A_{i+1} = \langle \Sigma, q_0, Q \cup Q^3 \setminus q_0^3, F, \delta \cup \delta^3_{[q_0^3 \leftarrow q_j]} \rangle$, *and* $\mathcal{I}_{i+1} = \mathcal{I}_i \cup \{(p^k, q^k) \mid p^k \in P_c, k \in \{1, 2, 3\}\}$, *where* $q^1 = q$ *and* $q^2 = q^3 = q_j$.

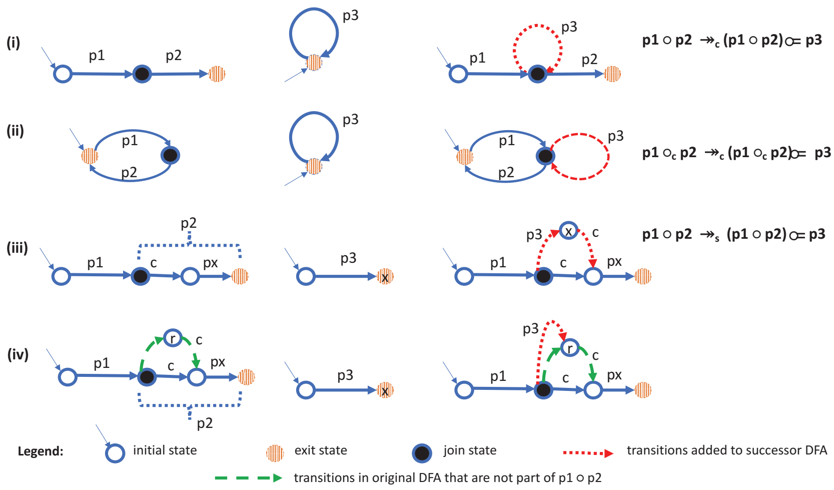

**Fig. 3.** Structure of DFA after applying rule of type 2 or type 3

Example applications of rule (2) are shown in Figures 3(i) and 3(ii).

We also wish to graft a non-circular pattern $p^3$ between $p^1$ and $p^2$, but this time we must avoid connecting the exit state $q^3_X$ to $q_j$ lest we loop over $p^3$ multiple times. We therefore replicate the outgoing transitions of $q_j$ in $p^1 \circ p^2$ to the inserted state $q^3_X$ so that they may act as the connections back into the DFA.

**Definition 17 (Rules of type (3)).** *A rule* $p \twoheadrightarrow_s (p^1 \circ p^2) \subseteq p^3$ *may extend* $\langle A_i, \mathcal{I}_i\rangle$ *at the join state* $q_j = \mathrm{join}(p, q, Ai)$ *of any instance* $(p, q) \in \mathcal{I}_i$*, provided* $\mathrm{def}(q_j) \cap \mathrm{def}(q^3_0) = \emptyset$*. This creates* $\langle A_{i+1}, \mathcal{I}_{i+1}\rangle$ *as follows:* $A_{i+1} = \langle \Sigma, q_0, Q \cup Q^3 \setminus q^3_0, F, \delta \cup \delta^3_{[q^3_0 \leftarrow q_j]} \cup C\rangle$ *where* $C = \{\ (q^3_X, \sigma, \delta(q_j, \sigma)) \mid\ \sigma \in \mathrm{def}(p^2, q^2_0)\}$*, and* $\mathcal{I}_{i+1} = \mathcal{I}_i \cup \{(p^k, q^k) \mid p^k \in P_c, k \in \{1, 2, 3\}\}$ *where* $q^1 = q$ *and* $q^2 = q^3 = q_j$*.*

We call $C$ the *connecting transitions*. We depict this rule application in example in Fig. 3 (iii), in which a member of $C$ is labeled 'c'.

Multiple applications of rules of type (3) to the same instance $\hat{p}$ will create several equivalent states in the resulting DFAs, as all of their exit states will have the same connecting transitions. These states are merged in a minimized representation, as depicted in Diagram (iv) of Figure 3.

We write $A \in G(\mathbf{P})$ if there exists a sequence of enabled DFAs derived from $\mathbf{P}$ s.t. $A = A_i$ for some $A_i$ in this sequence.

**Definition 18 (Language of a PRS).** *The language of a PRS* $\mathbf{P}$ *is the union of the languages of the DFAs it can generate:* $L(\mathbf{P}) = \cup_{A \in G(\mathbf{P})} L(A)$*.*

## 4.1 Examples

*Example 1:* Let $p^1$ and $p^2$ be the patterns accepting 'a' and 'b' respectively. Consider the PRS $R_{ab}$ with rules, $\bot \twoheadrightarrow p^1 \circ p^2$ and $p^1 \circ p^2 \twoheadrightarrow_s (p^1 \circ p^2) \subseteq (p^1 \circ p^2)$.

This PRS creates only one sequence of DFAs. Once the first rule creates the initial DFA, by continuously applying the second rule we obtain the infinite sequence of DFAs each satisfying $L(A_i) = \{a^j b^j : 1 \leq j \leq i\}$, and so $L(R_{ab}) = \{a^i b^i : i > 0\}$. Figure 2(i) presents $A_1$, while $A_2$ and $A_3$ appear in Figure 4(i). We can substitute any non-circular patterns for $p^1$ and $p^2$, creating the language $\{x^i y^i : i > 0\}$ for any non-circular pattern regular expressions $x$ and $y$.

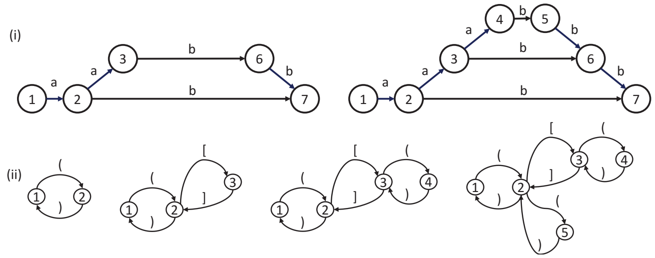

**Fig. 4.** DFA sequences for $R_{ab}$ and $R_{Dyck2}$

*Example 2:* Let $p^1$,$p^2$,$p^4$, and $p^5$ be the non-circular patterns accepting '(', ')', '[', and ']' respectively. Let $p^3 = p^1 \circ_c p^2$ and $p^6 = p^4 \circ_c p^5$. Let $R_{Dyck2}$ be the PRS containing rules $\bot \twoheadrightarrow p^3$, $\bot \twoheadrightarrow p^6$, $p^3 \twoheadrightarrow_c (p^1 \circ_c p^2) \Join p^3$, $p^3 \twoheadrightarrow_c (p^1 \circ_c p^2) \Join p^6$, $p^6 \twoheadrightarrow_c (p^4 \circ_c p^5) \Join p^3$, and $p^6 \twoheadrightarrow_c (p^4 \circ_c p^5) \Join p^6$. $R_{Dyck2}$ defines the Dyck language of order 2. Figure 4 (ii) shows one of its possible DFA-sequences.

## 5 PRS Inference Algorithm

A PRS can generate a sequence of DFAs defining, in the limit, a context-free language. We are now interested in inverting this process: given a sequence of DFAs generated by a PRS **P**, can we reconstruct **P**? Coupled with an $L^*$ extraction of DFAs from a trained RNN, solving this problem will enable us to extract a PRS from an RNN – provided the extraction follows a PRS (as we often find it does).

We present an algorithm for this problem, and show its correctness. In practice the DFAs we are given are not "perfect"; they contain noise that deviates from the PRS. We therefore augment this algorithm, allowing it to operate smoothly even on imperfect DFA sequences created from RNN extraction.

In the following, for each pattern instance $\hat{p}$ in $A_i$, we denote by $p$ the pattern that it is an instance of. We use similar notation $\hat{p}^1, \hat{p}^2$, and $\hat{p}^I$ to refer to specific instances of patterns $p^1, p^2$ and $p^I$. Additionally, for each consecutive DFA pair $A_i$ and $A_{i+1}$, we refer by $\hat{p}^3$ to the new pattern instance in $A_{i+1}$.

*Main steps of inference algorithm.* Given a sequence of DFAs $S = A_1 \cdots A_n$, the algorithm infers $\mathbf{P} = \langle \Sigma, P, P_c, R \rangle$ in the following stages:

1. Discover the initial pattern instance $\hat{p}^I$ in $A_1$. Insert $p^I$ into $P$ and mark $\hat{p}^I$ as enabled. Insert the rule $\bot \to p^I$ into $R$.

2. For $i, 1 \leq i \leq n-1$:
   (a) Discover the new pattern instance $\hat{p}^3$ in $A_{i+1}$ that extends $A_i$.
   (b) If $\hat{p}^3$ starts at the state $q_0$ of $A_{i+1}$, then it is an application of a rule of type (1). Insert $p^3$ into $P$, mark $\hat{p}^3$ as enabled, and add $\bot \twoheadrightarrow p^3$ to $R$.
   (c) Otherwise ($\hat{p}^3$ does not start at $q_0$), find the unique enabled pattern $\hat{p} = \hat{p}^1 \odot \hat{p}^2$ in $A_i$ s.t. $\hat{p}^3$'s initial state $q$ is the join state of $\hat{p}$. Add $p^1, p^2$, and $p^3$ to $P$, $p$ to $P_c$, and mark $\hat{p}^1$,$\hat{p}^2$, and $\hat{p}^3$ as enabled. If $\hat{p}^3$ is non-circular, add $p \twoheadrightarrow_s (p^1 \circ p^2) \propto p^3$ to $R$; otherwise add $p \twoheadrightarrow_c (p^1 \odot p^2) \propto p^3$.

3. Define $\Sigma$ to be the set of symbols used by the patterns $P$.

We now elaborate on how we determine the patterns $\hat{p}^I$, $\hat{p}^3$, and $\hat{p}$.

**Discovering new patterns $\hat{p}^I$ and $\hat{p}^3$** $A_1$ provides an initial pattern $p^I$. For subsequent DFAs, we need to identify which states in $A_{i+1} = \langle \Sigma, q_0', Q', F', \delta' \rangle$ are 'new' relative to $A_i = \langle \Sigma, q_0, Q, F, \delta \rangle$. From the PRS definitions, we know that there is a subset of states and transitions in $A_{i+1}$ that is isomorphic to $A_i$:

**Definition 19.** *(Existing states and transitions) For every $q' \in Q'$, we say that $q'$* exists *in $A_i$ with* parallel state *$q \in Q$ iff there exists a sequence $w \in \Sigma^*$ such that $q = \hat{\delta}(q_0, w)$, $q' = \hat{\delta}'(q_0, w)$, and neither is a sink reject state. Additionally, for every $q_1', q_2' \in Q'$ with parallel states $q_1, q_2 \in Q$, we say that $(q_1', \sigma, q_2') \in \delta'$* exists *in $A_i$ iff $(q_1, \sigma, q_2) \in \delta$. We denote $A_{i+1}$'s existing states and transitions by $Q_E \subseteq Q'$ and $\delta_E \subseteq \delta'$, and the* new *ones as $Q_N = Q' \setminus Q_E$ and $\delta_N = \delta' \setminus \delta_E$.*

By construction of PRSs, each state in $A_{i+1}$ has at most one parallel state in $A_i$, which can be found in one simultaneous traversal of the two DFAs.

The new states and transitions form a new pattern instance $\hat{p}$ in $A_{i+1}$, excluding its initial and possibly its exit state. The initial state of $\hat{p}$ is the existing state $q_s' \in Q_E$ that has outgoing new transitions. The exit state $q_X'$ of $\hat{p}$ is identified by the *Exit State Discovery* algorithm:

1. If there exists a $(q, \sigma, q_s') \in \delta_N$, then $\hat{p}$ is circular: $q_X' = q_s'$. (Fig. 3(i), (ii)).
2. Otherwise, $\hat{p}$ is non-circular. If it is the first (with respect to $S$) non-circular pattern grafted onto $q_s'$, then $q_X'$ is the unique new state whose transitions into $A_{i+1}$ are the *connecting* transitions from Definition 17 (Fig. 3 (iii)).
3. If there is no such state, then $\hat{p}$ is not the first non-circular pattern grafted onto $q_s'$, and $q_X'$ is the unique existing state $q_X' \neq q_s'$ with new incoming transitions. (Fig. 3(iv)).

Finally, the new pattern instance is $p = \langle \Sigma, q_s', Q_p, q_X', \delta_p \rangle$, where $Q_p = Q_N \cup \{q_s', q_X'\}$ and $\delta_p$ is the restriction of $\delta_N$ to the states of $Q_p$.

**Discovering the pattern $\hat{p}$ (step 2c)** In [27] we show that no two enabled pattern instances in a DFA can share a join state, that if they share any non-edge states, then one is contained in the other, and finally that a pattern's join states is never one of its edge states. This makes finding $\hat{p}$ straightforward: denoting $q_j$

as the parallel of $\hat{p}^3$'s initial state in $A_i$, we seek the enabled composite pattern instance $(p, q) \in \mathcal{I}_i$ for which $\text{join}(p, q, A_i) = q_j$. If none is present, we seek the only enabled instance $(p, q) \in \mathcal{I}_i$ that contains $q_j$ as a non-edge state, but is not yet marked as a composite. (Note that if two enabled instances share a non-edge state, then the containing one is already marked as a composite: otherwise we would not have found and enabled the other).

In [27] we define the concept of a *minimal generator* and prove the following:

**Theorem 1.** *Let $A_1, A_2, ...A_n$ be a finite sequence of DFAs that has a minimal generator* $\mathbf{P}$. *Then the PRS Inference Algorithm will discover* $\mathbf{P}$.

### 5.1 Deviations from the PRS framework

Given a sequence of DFAs generated by the rules of PRS $\mathbf{P}$, the inference algorithm given above will faithfully infer $\mathbf{P}$. In practice however, we want to apply the algorithm to a sequence of DFAs extracted from a trained RNN using the $L^*$ algorithm (as in [26]). Such a sequence may contain noise: artifacts from an imperfectly trained RNN, or from the behavior of $L^*$ . The major deviations are incorrect pattern creation, simultaneous rule applications, and slow initiation.

*Incorrect pattern creation* Whether due to inaccuracies in the RNN classification, or as artifacts of the $L^*$ process, incorrect patterns are often inserted into the DFA sequence. Fortunately, these patterns rarely repeat, and so we can discern between them and 'legitimate' patterns using a *voting* and *threshold* scheme.

The *vote* for each discovered pattern $p \in P$ is the number of times it has been inserted as the new pattern between a pair of DFAs $A_i, A_{i+1}$ in $S$. We set a *threshold* for the minimum vote a pattern needs to be considered valid, and only build rules around the connection of valid patterns onto the join states of other valid patterns. To do this, we modify the flow of the algorithm: before discovering rules, we first filter invalid patterns by splitting step 2 into two phases. *Phase 1:* Mark all the inserted patterns between each pair of DFAs, and compute their votes. Add to $P$ those whose vote is above the threshold. *Phase 2:* Consider each DFA pair $A_i, A_{i+1}$ in order. If the new pattern in $A_{i+1}$ is valid, and its initial state's parallel state in $A_i$ also lies in a valid pattern, then synthesize the rule according to the original algorithm. If a pattern is discovered to be composite, add its composing patterns to $P$.

As almost every DFA sequence produced by our method has some noise, the voting scheme greatly extended the reach of our algorithm.

*Simultaneous rule applications* In the theoretical framework, $A_{i+1}$ differs from $A_i$ by applying a *single* PRS rule, and therefore $q'_s$ and $q'_X$ are uniquely defined. $L^*$ however does not guarantee such minimal increments between DFAs. In particular, it may apply multiple PRS rules between two subsequent DFAs, extending $A_i$ with several patterns. To handle this, we expand the initial and exit state discovery methods given above.

1. Mark the new states and transitions $Q_N$ and $\delta_N$ as before.

2. Identify the *set* of new pattern instance initial states (*pattern heads*): the set $H \subseteq Q' \setminus Q_N$ of states in $A_{i+1}$ with outgoing new transitions.
3. For each pattern head $q' \in H$, compute the *relevant* sets $\delta_{N|q'} \subseteq \delta_N$ and $Q_{N|q'} \subseteq Q_N$ of new transitions and states: the members of $\delta_N$ and $Q_N$ that are reachable from $q'$ *without passing through any existing transitions.*
4. For each $q' \in H$, restrict to $Q_{N|q'}$ and $\delta_{N|q'}$ and compute $q'_X$ and $p$ as before.

If $A_{i+1}$'s new patterns have no overlap and do not create an ambiguity around join states, then they may be handled independently and in arbitrary order. They are used to discover rules and then enabled, as in the original algorithm.

Simultaneous but dependent rule applications – such as inserting a pattern and then grafting another onto its join state – are more difficult to handle, as it is not always possible to determine which pattern was grafted onto which. However, there is a special case which appeared in several of our experiments (examples L13 ad L14 of Section 7) for which we developed a technique as follows.

Suppose we discover a rule $r_1 : p_0 \twoheadrightarrow_s (p_l \circ p_r) \propto p$ and $p$ contains a cycle $c$ around some internal state $q_j$. If later another rule inserts a pattern $p_n$ at the state $q_j$, we understand that $p$ is in fact a composite pattern, with $p = p_1 \circ p_2$ and join state $q_j$. However, as patterns do not contain cycles at their edge states, $c$ cannot be a part of either $p_1$ or $p_2$. We conclude that the addition of $p$ was in fact a simultaneous application of two rules: $r'_1 : p_0 \twoheadrightarrow_s (p_l \circ p_r) \propto p'$ and $r_2 : p' \twoheadrightarrow_c (p_1 \circ p_2) \propto c$, where $p'$ is $p$ without the cycle $c$, and update our PRS and our DFAs' enabled pattern instances accordingly. The case when $p$ is circular and $r_1$ is of rule type (2) is handled similarly.

*Slow initiation* Ideally, $A_1$ directly supplies an initial rule $\bot \twoheadrightarrow p^I$ to our PRS. In practice, the first few DFAs generated by $L^*$ have almost random structure. We solve this by leaving discovery of the initial rules to the *end* of the algorithm, at which point we have a set of 'valid' patterns that we are sure are part of the PRS. From there we examine the *last* DFA $A_n$ generated in the sequence, note all the enabled instances $(p^I, q_0)$ at its initial state, and generate a rule $\bot \twoheadrightarrow p^I$ for each of them. This technique has the weakness that it will not recognise patterns $p^I$ that do not also appear as extending patterns $p_3$ elsewhere in the sequence, unless the threshold for patterns is minimal.

## 6 Converting a PRS to a CFG

We present an algorithm to convert a given PRS to a context free grammar (CFG), making the rules extracted by our algorithm more accessible.

*A restriction:* Let $\mathbf{P} = \langle \Sigma, P, P_c, R \rangle$ be a PRS. For simplicity, we restrict the PRS so that every pattern $p$ can only appear on the LHS of rules of type (2) or only on the LHS of rules of type (3) but cannot only appear on the LHS of both types of rules. Similarly, we assume that for each rule $\bot \rightarrow p_I$, the RHS patterns $p_I$ are all circular or non-circular. This restriction is natural: all of the examples in Sections 4.1 and 7.3 conform to it. Still, in [27] we show how to remove this restriction.

We create a CFG $G = \langle \Sigma, N, S, Prod \rangle$. $\Sigma$ is the same alphabet of $\mathbf{P}$ and we take $S$ as a special start symbol. For every pattern $p \in P$, let $G_p = \langle \Sigma_p, N_p, Z_p, Prod_p \rangle$ be a CFG describing $L(p)$. Let $P_Y \subseteq P_C$ be those composite patterns that appear on the LHS of a rule of type (2). Create the non-terminal $C_S$ and for each $p \in P_Y$, create an additional non-terminal $C_p$. We set $N = \{S, C_S\} \bigcup_{p \in P} \{N_p\} \bigcup_{p \in P_Y} \{C_p\}$.

Let $\bot \twoheadrightarrow p_I$ be a rule in $\mathbf{P}$. If $p_I$ is non-circular, create a production $S ::= Z_{p_I}$. If $p_I$ is circular, create the productions $S ::= S_C$, $S_C ::= S_C S_C$ and $S_C ::= Z_{p_I}$. For each rule $p \twoheadrightarrow_s (p_1 \circ p_2) \propto p_3$ create a production $Z_p ::= Z_{p_1} Z_{p_3} Z_{p_2}$. For each rule $p \twoheadrightarrow_c (p_1 \circ p_2) \propto p_3$ create productions $Z_p ::= Z_{p_1} C_p Z_{p_2}$, $C_p ::= C_p C_p$, and $C_p ::= Z_{p_3}$. Let $Prod'$ be the all the productions defined by the above process. We set $Prod = \{ \bigcup_{p \in P} Prod_p \} \cup Prod'$.

**Theorem 2.** *Let $G$ and $\mathbf{P}$ be as above. Then $L(\mathbf{P}) = L(G)$.*

The proof is given in the extended version of this paper [27].

*Expressibility* Every RE-Dyck language (Section 2.2) can be expressed by a PRS, but the converse is not true; RE-Dyck languages nest delimiters arbitrarily, while PRS grammars may not. For instance, language L12 of Section 7.3 is not a Dyck language. Meanwhile, not every CFL can be expressed by a PRS [27].

*Succinctness* The construction above does not necessarily yield a minimal CFG $G$. For a PRS defining the Dyck language of order 2 – which can be expressed by a CFG with 4 productions and 1 non-terminal – our construction yields a CFG with 10 non-terminals and 12 productions. In this case, and often in others, we can recognise and remove the spurious productions from the generated grammar.

## 7 Experimental results

### 7.1 Methodology

We test the algorithm on several PRS-expressible context free languages, attempting to extract them from trained RNNs using the process outlined in Figure 1. For each language, we create a probabilistic CFG generating it, train an RNN on samples from this grammar, extract a sequence of DFAs from the RNN, and apply our PRS inference algorithm. Finally, we convert the extracted PRS back to a CFG, and compare it to our target CFG.

In all of our experiments, we use a vote-threshold s.t. patterns with less than 2 votes are not used to form any PRS rules (Section 5.1). Using no threshold significantly degraded the results by including too much noise, while higher thresholds often caused us to overlook correct patterns and rules.

### 7.2 Generating a sequence of DFAs

We obtain a sequence of DFAs for a given CFG using only positive samples[11,1] by training a *language-model RNN* (LM-RNN) on these samples and then extracting DFAs from it with the aid of the $L^*$ algorithm [2], as described in [26]. To apply $L^*$ we must treat the LM-RNN as a binary classifier. We set an 'acceptance threshold' $t$ and define the RNN's language as the set of sequences $s$ satisfying: 1. the RNN's probability for an end-of-sequence token after $s$ is greater than $t$, and 2. at no point during $s$ does the RNN pass through a token with probability $< t$. This is identical to the concept of *locally $t$-truncated support* defined in [13].

To create the samples for the RNNs, we write a weighted version of the CFG, in which each non-terminal is given a probability over its rules. We then take $N$ samples from the weighted CFG according to its distribution, split them into train and validation sets, and train an RNN on the train set until the validation loss stops improving. In our experiments, we used $N = 10,000$. For our languages, we used very small 2-layer LSTMs: hidden dimension 10 and input dimension 4.

In some cases, especially when all of the patterns in the rules are several tokens long, the extraction of [26] terminates too soon: neither $L^*$ nor the RNN abstraction consider long sequences, and equivalence is reached between the $L^*$ hypothesis and the RNN abstraction despite neither being equivalent to the 'true' language of the RNN. In these cases we push the extraction a little further using two methods: first, if the RNN abstraction contains only a single state, we make an arbitrary initial refinement by splitting 10 hidden dimensions, and restart the extraction. If this is also not enough, we sample the RNN according to its distribution, in the hope of finding a counterexample to return to $L^*$ . The latter approach is not ideal: sampling the RNN may return very long sequences, effectively increasing the next DFA by many rule applications. We place a time limit of $1,000$ seconds ($\sim 17$ minutes) on the extraction.

### 7.3 Languages

We experiment on 15 PRS-expressible languages $L_1 - L_{15}$, grouped into 3 classes:

1. Languages of the form $\mathtt{X}^n\mathtt{Y}^n$, for various regular expressions `X` and `Y`. In particular, the languages $L_1$ through $L_6$ are $\mathtt{X}_i^n\mathtt{Y}_i^n$ for: $(\mathtt{X}_1,\mathtt{Y}_1)$=`(a,b)`, $(\mathtt{X}_2,\mathtt{Y}_2)$=`(a|b,c|d)`, $(\mathtt{X}_3,\mathtt{Y}_3)$=`(ab|cd,ef|gh)`, $(\mathtt{X}_4,\mathtt{Y}_4)$=`(ab,cd)`, $(\mathtt{X}_5,\mathtt{Y}_5)$=`(abc,def)`, and $(\mathtt{X}_6,\mathtt{Y}_6)$=`(ab|c,de|f)`.
2. Dyck and RE-Dyck languages. In particular, languages $L_7$ through $L_9$ are the Dyck languages of order 2 through 4, and $L_{10}$ and $L_{11}$ are RE-Dyck languages of order 1 with the delimiters $(\mathtt{L}_{10},\mathtt{R}_{10})$=`(abcde,vwxyz)` and $(\mathtt{L}_{11},\mathtt{R}_{11})$=`(ab|c,de|f)`.
3. Variations of the Dyck languages. $L_{12}$ is the language of alternating single-nested delimiters, generating only sequences of the sort `([([])])` or `[([])]`. $L_{13}$ and $L_{14}$ are Dyck-1 and Dyck-2 with additional neutral tokens `a,b,c` that may appear multiple times anywhere in the sequence. $L_{15}$ is like $L_{13}$ except that the neutral additions are the token `d` and the sequence `abc`, eg: `(abc()())d` is in $L_{15}$, but `a(bc()())d` is not.

| LG | DFAs | Init Pats | Final Pats | Min/Max Votes | CFG Correct | LG | DFAs | Init Pats | Final Pats | Min/Max Votes | CFG Correct |
|---|---|---|---|---|---|---|---|---|---|---|---|
| $L_1$ | 18 | 1 | 1 | 16/16 | Correct | $L_9$ | 30 | 6 | 4 | 5/8 | Correct |
| $L_2$ | 16 | 1 | 1 | 14/14 | Correct | $L_{10}$ | 6 | 2 | 1 | 3/3 | Correct |
| $L_3$ | 14 | 6 | 4 | 2/4 | Incorrect | $L_{11}$ | 24 | 6 | 3 | 5/12 | Incorrect |
| $L_4$ | 8 | 2 | 1 | 5/5 | Correct | $L_{12}$ | 28 | 2 | 2 | 13/13 | Correct |
| $L_5$ | 10 | 2 | 1 | 7/7 | Correct | $L_{13}$ | 9 | 6 | 1 | 2/2 | Correct |
| $L_6$ | 22 | 9 | 4 | 3/16 | Incorrect | $L_{14}$ | 17 | 5 | 2 | 5/7 | Correct |
| $L_7$ | 24 | 2 | 2 | 11/11 | Correct | $L_{15}$ | 13 | 6 | 4 | 3/6 | Incorrect |
| $L_8$ | 22 | 5 | 4 | 2/9 | Partial | | | | | | |

**Table 1.** Results of experiments on DFAs extracted from RNNs

### 7.4 Results

Table 1 shows the results. The 2nd column shows the number of DFAs extracted from the RNN. The 3rd and 4th columns present the number of patterns found by the algorithm before and after applying vote-thresholding to remove noise. The 5th column gives the minimum and maximum votes received by the final patterns (we count only patterns introduced as a new pattern $p^3$ in some $A_{i+1}$). The 6th column notes whether the algorithm found a correct CFG, according to our manual inspection. For languages where our algorithm only missed or included 1 or 2 valid/invalid productions, we label it as partially correct.

*Alternating Patterns* Our algorithm struggled on the languages $L_3$, $L_6$, and $L_{11}$, which contained patterns whose regular expressions had alternations (such as `ab|cd` in $L_3$, and `ab|c` in $L_6$ and $L_{11}$). Investigating their DFA sequences uncovered the that the $L^*$ extraction had 'split' the alternating expressions, adding their parts to the DFAs over multiple iterations. For example, in the sequence generated for $L_3$, `ef` appeared in $A_7$ without `gh` alongside it. The next DFA corrected this mistake but the inference algorithm could not piece together these two separate steps into a single rule. It will be valuable to expand the algorithm to these cases.

*Simultaneous Applications* Originally our algorithm failed to accurately generate $L_{13}$ and $L_{14}$ due to simultaneous rule applications. However, using the technique described in Section 5.1 we were able to correctly infer these grammars. However, more work is needed to handle simultaneous rule applications in general.

Additionally, sometimes a very large counterexample was returned to $L^*$ , creating a large increase in the DFAs: the 9$^{\text{th}}$iteration of the extraction on $L_3$ introduced almost 30 new states. The algorithm does not manage to infer anything meaningful from these nested, simultaneous applications.

*Missing Rules* For the Dyck languages $L_7 - L_9$, the inference algorithm was mostly successful. However, due to the large number of possible delimiter combinations, some patterns and nesting relations did not appear often enough in the DFA

sequences. As a result, for $L_8$, some productions were missing in the generated grammar. $L_8$ also created one incorrect production due to noise in the sequence (one erroneous pattern was generated two times,passing the threshold).

*RNN Noise* In $L_{15}$, the extracted DFAs for some reason always forced that a single character `d` be included between every pair of delimiters. Our inference algorithm of course maintained this peculiarity. It correctly allowed the allowed optional embedding of "abc" strings. But due to noisy (incorrect) generated DFAs, the patterns generated did not maintain balanced parenthesis.

## 8 Related work

*Training RNNs to recognize Dyck Grammars.* Recently there has been a surge of interest in whether RNNs can learn Dyck languages [5,19,21,28]. While these works report very good results on learning the language for sentences of similar distance and depth as the training set, with the exception of [21], they report significantly lower accuracy for out-of-sample sentences.

Among these, Sennhauser and Berwick [19] use LSTMs, and show that in order to keep the error rate within a 5 percent tolerance, the number of hidden units must grow exponentially with the distance or depth of the sequences (though Hewitt et. al. [13] find much lower theoretical bounds). They conclude that LSTMs do not learn rules, but rather statistical approximations. Bernardy [5] experimented with various RNN architectures, finding in particular that the LSTM has more difficulty in predicting closing delimiters in the middle of a sentence than at the end. Based on this, he conjectures that the RNN is using a counting mechanism, but has not truly learnt the Dyck language (its CFG). For the simplified task of predicting only the final closing delimiter of a legal sequence, Skachkova, Trost and Klakow [21] find that LSTMs have nearly perfect accuracy across words with large distances and embedded depth.

Yu, Vu and Kuhn [28] compare the three works above, and note that the task of predicting only the closing bracket of a balanced Dyck word is not sufficient for checking if an RNN has learnt the language, as it can be computed by only a counter. In their experiments, they present a prefix of a Dyck word and train the RNN to predict the next valid closing bracket. They experiment with an LSTM using 4 different models, and show that the generator-attention model [17] performs the best, and is able to generalize quite well at the tagging task . However, they find that it degrades rapidly with out-of-domain tests. They also conclude that RNNs do not really learn the Dyck language. These experimental results are reinforced by the theoretical work in [13], who remark that no finite precision RNN can learn a Dyck language of unbounded depth, and give precise bounds on the memory required to learn a Dyck language of bounded depth.

Despite these findings, our algorithm nevertheless extracts a CFG from a trained RNN, discovering rules based on DFAs synthesized from the RNN using the algorithm in [26]. Because we can use a short sequence of DFAs to extract the rules, and because the first DFAs in the sequence describe Dyck words with increasing but limited distance and depth, we are often able to extract the CFG perfectly even when the RNN does not generalize well. Moreover, we show that our approach works with more complex types of delimiters, and on Dyck languages with expressions between delimiters.

*Extracting DFAs from RNNs.* There have been many approaches to extract higher level representations from a neural network (NN), both to facilitate comprehension and to verify correctness. One of the oldest approaches is to extract rules from a NN [24,12]. In particular, several works attempt to extract FSAs from RNNs [18,15,25]. We base our work on [26]. Its ability to generate sequences of DFAs providing increasingly better approximations of the CFL is critical to our method.

There has been less research on extracting a CFG from an RNN. One exception is [23], where they develop a Neural Network Pushdown Automata (NNPDA) framework, a hybrid system augmenting an RNN with external stack memory. They show how to extract a push-down automaton from an NNPDA, however, their technique relies on the PDA-like structure of the inspected architecture. In contrast, we extract CFGs from RNNs without stack augmentation.

*Learning CFGs from samples.* There is a wide body of work on learning CFGs from samples. An overview is given in [10] and a survey of work for grammatical inference applied to software engineering tasks can be found in [22].

Clark et. al. studies algorithms for learning CFLs given only positive examples [11]. In [7], Clark and Eyraud show how one can learn a subclass of CFLs called *CF substitutable* languages. There are many languages that can be expressed by a PRS but are not substitutable, such as $x^n b^n$. However, there are also substitutable languages that cannot be expressed by a PRS ($wxw^R$ - see [27]). In [8], Clark, Eyraud and Habrard present Contextual Binary Feature Grammars. However, it does not include Dyck languages of arbitrary order. None of these techniques deal with noise in the data, essential to learning a language from an RNN.

## 9 Future Directions

Currently, for each experiment, we train the RNN on that language and then apply the PRS inference algorithm on a single DFA sequence generated from that RNN. Perhaps the most substantial improvement we can make is to extend our technique to learn from multiple DFA sequences. We can train multiple RNNs and generate DFA sequences for each one. We can then run the PRS inference algorithm on each of these sequences, and generate a CFG based upon rules that are found in a significant number of the runs. This would require care to guarantee that the final rules form a cohesive CFG. It would also address the issue that not all rules are expressed in a single DFA sequence, and that some grammars may have rules that are executed only once per word of the language.

Our work generates CFGs for generalized Dyck languages, but it is possible to generalize PRSs to express a greater range of languages. Work will then be needed to extend the PRS inference algorithm.
